# New-generation taxoid SB-T-1214 inhibits stem cell-related gene expression in 3D cancer spheroids induced by purified colon tumor-initiating cells

**DOI:** 10.1186/1476-4598-9-192

**Published:** 2010-07-14

**Authors:** Galina I Botchkina, Edison S Zuniga, Manisha Das, Yuan Wang, Hichao Wang, Shu Zhu, Anne G Savitt, Rebecca A Rowehl, Yan Leyfman, Jingfang Ju, Kenneth Shroyer, Iwao Ojima

**Affiliations:** 1 Department of Pathology, SUNY Stony Brook, NY, USA; 2 Institute of Chemical Biology & Drug Discovery, SUNY Stony Brook, NY, USA; 3 Department of Emergency Medicine, North Shore University Hospital, NYU School of Medicine Manhasset, NY, USA; 4 Department of Molecular Genetics and Microbiology, SUNY Stony Brook, NY, USA

## Abstract

**Background:**

Growing evidence suggests that the majority of tumors are organized hierarchically, comprising a population of tumor-initiating, or cancer stem cells (CSCs) responsible for tumor development, maintenance and resistance to drugs. Previously we have shown that the CD133^high^/CD44^high ^fraction of colon cancer cells is different from their bulk counterparts at the functional, morphological and genomic levels. In contrast to the majority of colon cancer cells expressing moderate levels of CD133, CD44 and CD166, cells with a high combined expression of CD133 and CD44 possessed several characteristic stem cell features, including profound self-renewal capacity *in vivo *and *in vitro*, and the ability to give rise to different cell phenotypes. The present study was undertaken for two aims: a) to determine stem cell-related genomic characteristics of floating 3D multicellular spheroids induced by CD133^high^/CD44^high ^colon cancer cells; and b) to evaluate CSC-specific alterations induced by new-generation taxoid SB-T-1214.

**Results:**

Selected CSC phenotype was isolated from three independent invasive colon cancer cell lines, HCT116, HT29 and DLD-1. A stem cell-specific PCR array assay (*SA*Biosciences) revealed that colonospheres induced by purified CD133^high^/CD44^high ^expressing cells display profound up-regulation of stem cell-related genes in comparison with their bulk counterparts. The FACS analysis has shown that the 3D colonospheres contained some minority cell populations with high levels of expression of Oct4, Sox2, Nanog and c-Myc, which are essential for stem cell pluripotency and self-renewal. Single administration of the SB-T-1214 at concentration 100 nM-1 μM for 48 hr not only induced growth inhibition and apoptotic cell death in these three types of colon cancer spheroids in 3D culture, but also mediated massive inhibition of the stem cell-related genes and significant down-regulation of the pluripotency gene expression. PCR array and FACS data were confirmed with western blotting. Importantly, viable cells that survived this treatment regimen were no longer able to induce secondary floating spheroids and exhibited significant morphological abnormalities.

**Conclusions:**

We report here that a new-generation taxoid SB-T-1214 possesses significant activity against colon cancer spheroids induced by and enriched with drug resistant tumorigenic CD133^high^/CD44^high ^cells and efficiently inhibited expression of the majority of stem cell-related genes. Our data indicates that the previously observed long-term efficacy of SB-T-1214 against drug resistant colon tumors *in vivo *may be explained by the down-regulation of multiple stem cell-related genes in the tumorigenic cell population, in addition to its known efficacy as a mitotic poison against proliferating cancer cells.

## Background

With a worldwide cumulative incidence rate of 9.4%, colorectal cancer is the second leading cause of cancer deaths when both sexes are combined [1]. Currently, anti-cancer drug development has a significantly lower success rate compared to other diseases [2], and this ineffectiveness of standard anti-cancer drugs has been attributed to the existence of relatively rare, highly drug resistant, quiescent or slow proliferating tumor-driving cells - cancer stem cells (CSCs). After the successful isolation and characterization of CSCs from all major types of human liquid and solid tumors [reviewed in ref. [3]] it became evident that CSCs are exclusively endowed with tumor-initiating capacity in the majority, if not all, cancer types, and are responsible for tumor sustaining, recurrence, metastasis and resistance to treatment. CSCs give rise to a hierarchy of actively proliferating, but progressively differentiating, tumor cells (committed progenitors), which contribute to the cellular heterogeneity of human cancers. Many types of CSCs, including tumor-initiating cells in brain [4,5], kidney [6], liver [7,8], colon [9,10] and pancreatic [11] carcinomas were isolated and enriched using the cell surface marker CD133, or prominin-1 [12] alone or in combination with some other markers. Another commonly used cell surface marker is CD44 [13-22]. This glycoprotein is involved in many cell-cell interactions, stemness and tumor development, in part via *β*-catenin and Wnt signaling activation of the CD44 gene transcription [23,24]. It was demonstrated earlier that the full range of CD44 alternatively spliced variants is widely expressed in normal and tumor colonic cells located in the crypt base [25], known as a colonic stem cell niche. Numerous studies have demonstrated that both CD133^+ ^and CD44^+ ^cells are highly tolerant to anti-cancer therapies [21,26-33], and moreover, the number of CSCs can be significantly increased after treatment [28,34-38]. The ratio of CD133^+ ^cells correlates with tumor aggressiveness, histologic grade and clinical outcome [5,39-42]. Similar data were reported for CD44-positive cells [21].

Since CSCs are naturally resistant to chemotherapy due to multiple mechanisms, including their relative quiescence, profound capacity for DNA repair, activation of the ATP-binding cassette (ABC) transporters that efflux many standard anticancer agents, resistance to apoptosis and others [43-45], it is conceivable that effective anti-cancer drugs must be specifically targeted toward CSCs, not only to bulk tumor cells. Colon cancer is inherently drug-resistant due to multiple mechanisms that are still poorly characterized, so both the stem cells and the variably differentiated cells that comprise the proliferative pool of the colorectal carcinoma can potentially contribute to chemotherapy tolerance. The CSCs are biologically distinct from differentiated normal and cancerous cells; therefore, the search for therapies that are specific for CSCs should be focused on differences in gene expression patterns between these cell types. Previously we have shown that several new-generation taxoids, which were developed as an attempt to improve widely used taxane-based anticancer agents [46], exhibited cytotoxicity 2-3 orders of magnitude higher than commonly used paclitaxel and docetaxel against drug resistant breast cancer cells overexpressing the Pgp efflux pump [47,48]. One of these taxoids, SB-T-1214, also exhibited high efficiency against colon cancer *in vivo*, inducing complete regression of drug-resistant colon tumor xenografts in all surviving mice with tumor growth delay up to 201 days [49]. Such promising antitumor activity of SB-T-1214 led us to suggest that this compound can specifically target tumor-specific CSCs by inhibiting some stemness-related signaling pathways and/or promoting their differentiation. Since CSC-enriched CD133^+ ^and CD44^+ ^cell populations are more resistant to conventional therapies than other more differentiated cells [21,26-33], it was important to test cytotoxic effects of SB-1214 against these cell phenotypes. Since CSCs represent a dynamic population with dual potential, self-renewal versus generation of the committed progenitors, which eventually will differentiate into all mature cell phenotypes, the isolated CSCs was cultured, tested and treated under conditions designed to retain their "stemness" and preclude differentiation to the bulk tumor cells. Recent discovery of the possibility to reprogram adult differentiated epithelial cells into induced pluripotent stem cells by introduction of several essential transcription factors (Oct4, Sox2, c-Myc and Klf4) that determine cell stemness/differentiation state and regulate stem cell self-renewal [50,51], makes these genes an important target for anticancer drug development. The present study was thus undertaken for two aims: a) to determine the stem cell-related genomic differences between CSC-enriched floating colonospheres grown from CD133^high^/CD44^high ^cells in comparison to their bulk counterparts using three independent colon cancer cell lines; and b) to evaluate CSC-specific alterations induced by new-generation taxoid SBT-1214.

## Results

**Figure 1:**
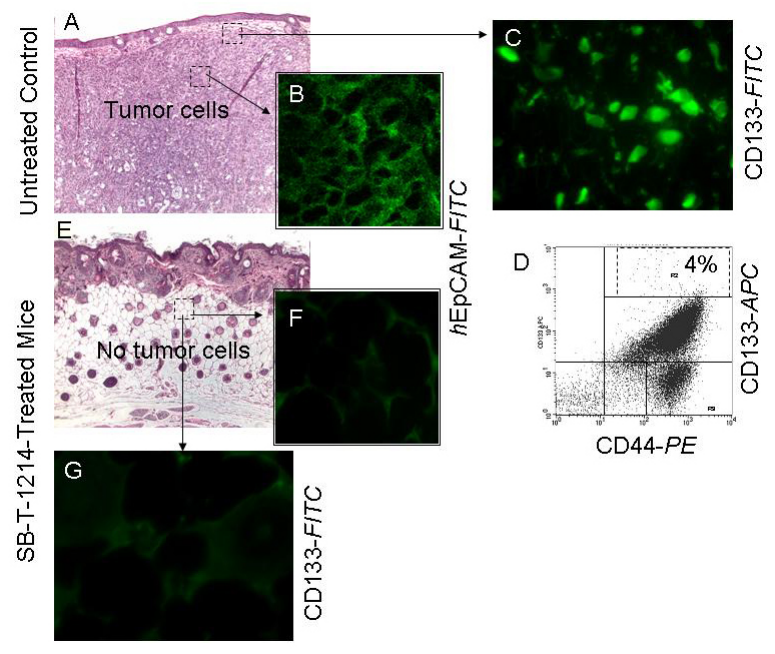
**Histopathological, immunohistochemical and FACS analyses of the mice tumor xenografts**. Tumors recovered from the control (vehicle treated; A-D) and SB-T-1214 treated (E-G) mice were sectioned and stained with hematoxylin and eosin (A, E), human epithelial adhesion molecule *h*EpCAM-*FITC*; B, F) and CD133-FITC (C, G). The tissue section from vehicle treated mouse show large area of living tumor positive for human EpCAM-*FITC *(B). Cluster of the CD133-positive cells from the outer tumor area (C). FACS analysis of the dissociated and immunomagnetically sorted for hEpCAM untreated mice tumor xenograft shows the presence of the minor population of cancer cells with high combined expression of CD133-*APC *and CD44-*PE *(dashed square; D). Complete loss of the tumor cells (E) and *h*EpCAM- (F) and CD133-positivity (G) after three consequents treatments with SB-T-1214.

A new-generation taxoid SB-T-1214 was evaluated for its efficacy against several drug-resistant human tumor xenografts, including colon tumors (Pgp+ DLD-1) in severe combined immune deficient (SCID) mice in our previous study [49]. The drug was administered intravenously in three doses 3 times using a 3-day regimen (q3d × 3, on day 5, 8, and 11), starting from day 5 after DLD-1 subcutaneous tumor implantation (results are summarized in Table 1). As anticipated, paclitaxel was ineffective against this highly drug-resistant (Pgp+) tumor at its optimal dose (60 mg/kg total dose). In contrast, SB-T-1214 has shown profound antitumor activity. The best result was obtained at 60 mg/kg total dose, 20 mg/kg × 3, wherein complete regression of the DLD-1 tumor was achieved in five of five mice (tumor growth delay was >201 days). Systemic toxicity profile has shown that there was only a 3-5% weight loss during the period of day 15 to day 20, and the drug was well tolerated by animals. Histopathological analysis of the hematoxylin and eosin stained tissue sections of the tumor xenografts recovered from the control (vehicle treated) mice revealed a large tumor areas with densely packed tumor cells (Figure 1A), which uniformly expressed membrane-bounded immunoreactivity for human epithelial cell adhesion molecule, *h*EpCAM (Figure 1B; *h*EpCAM-*FITC*; Biosource, CA, USA). Several small clusters of cells with high levels of CD133 expression were found predominantly within the outer areas of the tumors corresponding to the tumor invasive front (Figure 1C), and scattered CD133+ cells were detected across the entire tumor areas. Flow cytometry analysis of the dissociated and immunomagnetically sorted (MACS-*h*EpCAM) mice tumor xenografts confirmed the presence of a minor population (about 4%) of human cancer cells with high combined expression of the CD133 and CD44 (Figure 1D). After three consequent treatments with the SB-T-1214, we observed a complete reduction in tumor volume (Figure 1E). Residual tissues showed multiple inflammatory infiltrates and fibrosis (not shown), and they were negative for human EpCAM (F) and CD133 (G). Since SB-T-1214-treated mice did not show any presence of human cells, FACS analysis of the mice tissues was not performed.

**Table 1:** Antitumor effect of SBT-1214 delivered i.v. to SCID mice bearing PgP+ DLD-1 human colon tumor xenografts.

**Treatment**^**a**^	Sche-dule	Total Dose (mg/kg)	Dose/inj (mg/kg)	**Days to 600 mm**^**3**^	***P*value**^**b **^**(Control)**	Growth Delay (days)	**Toxicity**^**c**^	**Cured Mice per group**^**d**^
Control		0	0	17	---	---	0	0/10
Vehicle-tween Paclitaxel	q3d × 3 q3d × 3	0 60	0 20	16	.405 <.001	-1 8	0 0	0/4 0/5
SB-1214	q3d × 3	30	10	54	<.001	37	0	0/5
SB-1214	q3d × 3	60	20	>201	<.001	>150	0	5/5
SB-1214	q3d × 3	120	40	>201	.001	>150	2	3/5

^a^Treatment given IV to SCID mice on day 5 after DLD-1 human colon tumor implant, and continued as noted; all drugs formulated in Tween/EtOH

^b^Based on comparison of each group vs. control using the Cox-Mantel Test

^c^Number of animals who either died or lost greater than 20% body weight

^d^SCID mice with no palpable tumor at end of experiment day 167.

### Isolation, purification and propagation of colon CSC phenotype

**Figure 2:**
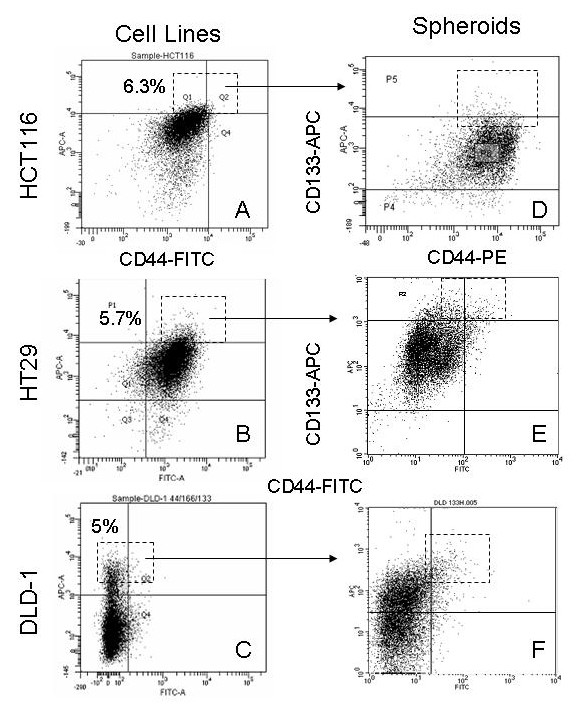
**The CD133^high^/CD44^high ^phenotypic cell population has high sphere-forming capacity in the three independent colon cancer cell lines HCT116, HT29 and DLD-1**. Cells grown at standard culture conditions possessed the minority phenotypic subpopulations with high combined levels of expression of CD133 and CD44 (left panel; outlined by dashed squares). The sphere-forming efficiency of these cells was 1 of 66 for HCT116 cell line, 1 of 175 for HT29, and 1 of 118 for DLD-1 cell line. In contrast, bulk cancer cells could induce only a few loose flat colonies under non-adherent culture conditions. Induced spheroids retained original cell phenotypes (right panel).

To study the effects of SB-T-1214 specifically targeted to colon CSCs, we have selected three independent colon cancer cell lines, including DLD-1, which was used in our previous *in vivo *studies of the antitumor activity of SB-T-1214, and two other highly invasive colon cancer cell lines, HCT116 and HT29. We have found that the vast majority (bulk) of all types of cells grown at standard adherent conditions expressed moderate levels of CD133 and CD44 (Figure 2; A-C). However, all three cell lines possessed minority cell populations with highest expression of CD133, which coincided with high expression of CD44 (CD133^high^/CD44^high^; red squares on A-C). Previously, we have characterized this phenotypic subpopulation in colon and prostate cancers by functional and genome-wide analysis, and demonstrated that these cells have profound self-renewing capacity *in vivo *and *in vitro*, and 3D spheroids induced by this cell phenotype possessed stem-like features in contrast to their bulk counterparts [19,20]. Even without additional purification, the acutely isolated CD133^high^/CD44^high ^cells derived from all three colon cancer cell lines possessed relatively high efficiency in forming dense floating multicellular spheroids in non-adherent cultures with serum-free medium (Figure 2; A-C), in contrast to their corresponding bulk counterparts, which produced a few loose flat colonies (not shown). The HCT116 spheroids revealed higher efficiency (one of 66 isolated cells induced dense 3D spheres) compared to HT29 (one of 175 cells) and DLD-1 (one of 118 cells). Dissociated spheroid cells retained an original cell phenotype and expressed all the studied commonly used stem cell surface markers, including CD133, CD44, CD166, *h*EpCAM, CD49b, and CD117 (CD133^high^/CD44^high ^population is shown on Figure 2; D-F, dashed squares). Immunohistochemical analysis revealed a minority cell population expressing high levels of nuclear *β*-catenin (not shown). Induced multicellular spheroids were further tested for the expression of stem cell-related genes and CSC-related activity of SB-T-1214 in comparison to their bulk counterparts using PCR array assay (*SA*Biosciences). However, these experiments required not only enriched CSCs, but also culture conditions which allow for retaining of the stem-like phenotype during drug treatment. Previously, we have found that additional purification of CD133^high^/CD44^high ^cell population by repeated sets of cell sorting followed by culturing at low cell density on type I collagen in serum-free stem cell medium led to significantly higher *in vivo *tumorigenic potential and sphere forming capacity of this cell phenotype [19,20]. In addition, the levels of expression of these markers and the ratios of CD133^high^/CD44^high ^cells in both mice tumor xenografts and colonospheres were also higher after transplantation of the purified cell populations. Our observations are in line with recent data showing that type I collagen indeed promotes the expression of a stem cell-like phenotype in human colorectal cancer and increases the expression of CD133 [52]. These approaches were used for growing 3D spheroids enriched with highly tumorigenic and clonogenic cells to be able to reveal genomic differences between these relatively rare cells in comparison to their much more numerous bulk counterparts. They were also used as target populations for studying CSC-specific drug effects.

### Cytotoxic effects of SB-T-1214 against CSC-enriched colon cancer cells

**Figure 3:**
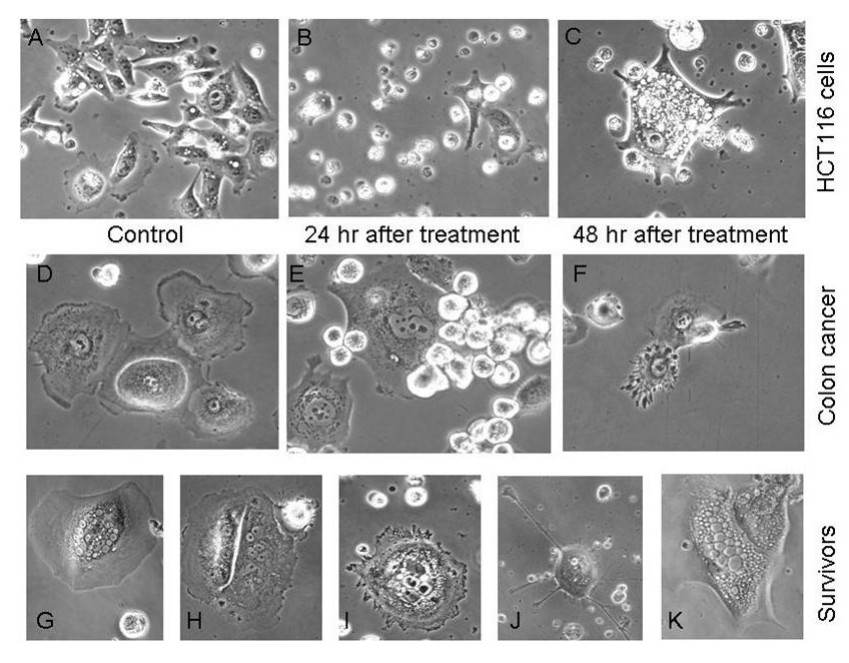
**Cytotoxic effects of SB-T-1214 on colon CSC-enriched cell populations grown on type I collagen-coated surfaces**. FACS sorted CD133^high^/CD44^high ^cells derived from colon cancer cell lines (HCT116 is shown) and peritoneal fluid of patient with metastatic colon cancer were plated for a short time on collagen type I-coated plates in the MSCB medium. Although a majority of these cells were killed after 48 hours incubation with 100 nM of SB-T-1214, some percentage of the cells survived treatment (A-C, D-F). The cells which survived treatment expressed multiple abnormalities, including greatly enlarged size (G-I, K), multiple nuclei (E, G-I), significant increase in the number of long (J), and knobby (F, I) projections and severe vacuolization (K). A-C: magnification 10×; D-K: magnification 40×.

The CSC-specific cytotoxic effects of SB-T-1214 were studied in two different settings which promote stemness phenotype: monolayer adherent to type I collagen cultures and three dimensional cultures of floating spheroids. In a first setting, the FACS sorted CD133^high^/CD44^high ^cells isolated from peritoneal fluid of metastatic colon cancer patients or from the established colon cancer cell lines were plated on the type I collagen-coated plates and cultured in the MSCB serum-free medium for 1-2 days before treatment. After 48 hours of incubation with the drug in concentration ranging from 100 nM-1 μM, a majority (89-96%) of all cell types underwent apoptosis [Figure 3; shown HCT116 cells (A-C); and peritoneal wash from metastatic colon cancer patient (D-F)]. Although about 4-11% of cells survived this treatment regimen, such cells displayed multiple abnormalities, including a greatly enlarged size (G-I, K), multiple nuclei (E, G-I), a significant increase in the number of long (J), and knobby (F, I) projections and severe vacuolization (K). Many cells displayed a clear sign of the mitotic catastrophe (G-I).

**Figure 4:**
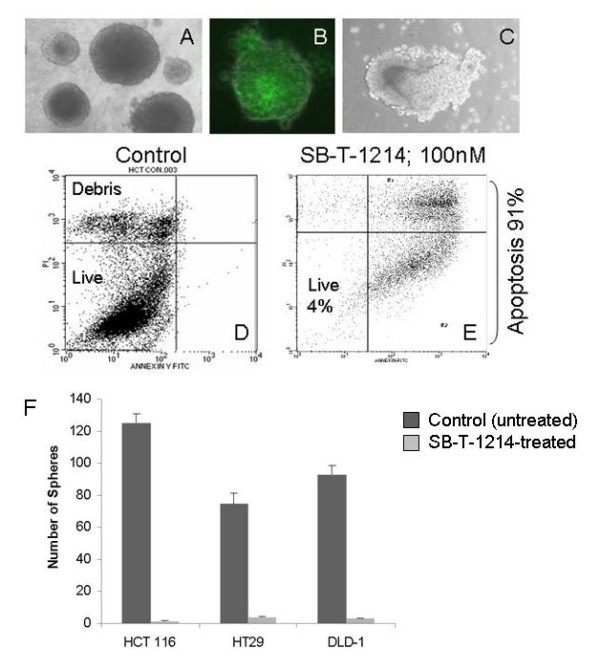
**Cytotoxic effects of the SB-T-1214 against colonospheres grown in 3D cultures**. Ten day-old colonospheres grown from FACS sorted CD133^high^/CD44^high ^HCT116 cells on the ultra-low attachment 6-well plates (Corning) were treated with 100 nM SB-T-1214 for 48 hours, then drug-containing medium was replaced with regular MSCBM and analyzed after 24 hours. Treatment with drug induced a profound loss of sphere integrity (A, C) and apoptosis in the majority of the sphere cells (*D-E; *FACS analysis with the annexin V-FITC and propidium iodide). The FITC-conjugated drug showed efficient penetration into spheroid after 30 min of incubation (B). To evaluate possible alterations in sphere-forming capacity, control and drug treated spheroids/cells were dissociated to a single cell suspension and an equal number of viable cells (400 cells) were plated on ULA 6-well plates in MSCB medium containing 10% Matrigel. The number of secondary spheroids was counted ten days after plating (F).

Next we have studied the SB-T-1214 cytotoxicity against colon cancer spheroids induced by CD133^high^/CD44^high ^cells in 3D cultures. Administration of 0.1-1 μM SB-T-1214 for 48 hours induced a loss of integrity of the floating spheroids (Figure 4, A-C) and apoptosis in more than 90% of the sphere cells (D, E). The FITC-conjugated drug revealed efficient penetration into spheroids (C; 30 min exposure). Most importantly, viable cells which survived this treatment regimen significantly lost the ability to form secondary spheroids, which indicates that colon CSC population was critically affected (Figure 4, F). Thus, 1000 of untreated HCT116 primary spheroid cells induced 125 ± 6 secondary spheroids, HT29 - 75 ± 7, and DLD-1 gave rise to 93 ± 6 secondary spheroids, whereas the SB-T-1214-treated dissociated spheroid cells produced only 1.5 ± 0.3, 4 ± 0.6, and 3 ± 0.4 secondary spheroids, correspondently (*P *< 0.01). After placement on type I collagen surfaces, cells that survived drug treatment, displayed profound morphological abnormalities similar to those described above (Figure 3, G-K)

### Alterations in the stemness-related gene expression profile induced by SB-T-1214

**Figure 5:**
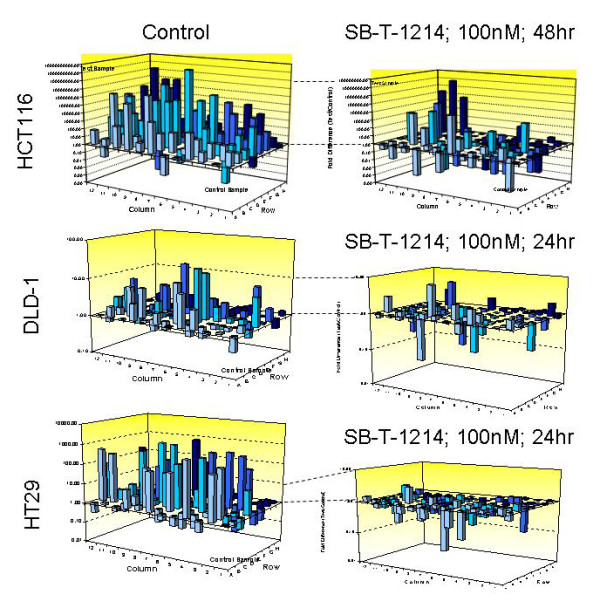
**Drug-induced alteration in the stem cell-related gene expression profiles (PCR Array assay)**. A majority of the stemness genes were up-regulated in floating spheroids grown from CD133^high^/CD44^high ^cells derived from HCT116, HT29 and DLD-1 cell lines in comparison with their corresponding bulk counterparts (left panel). Treatment of colonospheres with 100 nM SB-T-1214 induced significant down-regulation of a majority of the stemness genes (treated colonospheres were analyzed compared to untreated ones).

The CD133^high^/CD44^high ^cell populations derived from the three analyzed colon cancer cell lines were characterized with the stem cell pathway-specific PCR Array assay (*SA*Biosciences). Each array contains SYBR Green-based real-time PCR gene-specific assays for a set of 84 genes. Using filtering criteria of a 1.5 or greater fold-change in expression, we have analyzed differentially expressed genes in these three types of floating colonospheres compared to their bulk differentiated adherent counterparts, as well as before and after treatment with SB-T-1214. The most profound differences were observed in HCT116 spheroids grown from CD133^high^/CD44^high ^cells (Figure 5; left panel), which is in line with their higher sphere-forming and tumor-initiating capacities. About one-fourth of the analyzed stem cell-related genes, including Wnt and Notch pathway genes responsible for self-renew and cell cycle regulation, were commonly up-regulated in all types of spheroids, with significantly higher levels of expression in HCT116 ones. Thus, 6 of 6 analyzed genes responsible for stem cell self-renewal (*SOX1, SOX2, MYST1, MYST2, NEUROG2 *and *HSPA9*), and 3 of 5 genes regulating symmetrical/asymmetricasl cell division (*NOTCH1, NOTCH2 *and *PARD6A*) were significantly up-regulated in the HCT116 CD133/CD44-high colonospheres compared to their bulk counterparts. The most significantly up-regulated genes in HT29 spheroids were *ACAN, ALPI, APC, ASCL2, CCND2, CD3D, CD4, CD8A, CD8B, COL2A1, COL9A1, DHH, DLL3, DTX1 FGF1, GJA1, S100B, SOX2, T, TERT *and *WNT1; *and in DLD-1 spheroids *- ALDH1A1, ASCL2, CCND2, CD4, COL1A1, DLL1, DTX1, FGF1, GJA1, IGF1, JAG1, MME, NCAM1*, and *NOTCH1*.

Importantly, relatively low concentrations of SB-T-1214 (100 nM-1 μM for 24 or 48 hr) induced dramatic down-regulation of stemness in the majority of stem cell-related genes in all three types of colonospheres (Figure 5, right panel). The most significant drug-induced down-regulation of gene expression was detected: 1) in HCT116 colonospheres for *SOX1, RPL13A, BMP3, NEUROG2, GJB1, GJA1, ASCL2, CTNNA1, GDF2, ALPI, S100B, CD8B1, ACTB, CCND1, FGF1, PARD6A, DVL1, GDF3, ISL1, CD3D, MME, FGFR1, RB1, BMP1, AIN1, ALDH1A1, CD8A, PPARD, FZD1, NUMB, ABCG2*; 2) in HT29 colonospheres for *ACAN, ALPI, BMP3, CD3D, CD4, CD8A, CD8B, CDH2, COL2A1, COL9A1, DHH, DLL1, DLL3, DTX1, FGF1, FGF3, FZD1, GDF2, IGF1, MME, MYOD, NCAM1, NEUROG2, S100B, SOX2*, and *TERT*; 3) in DLD-1 colonospheres for *CD4, CDH2, COL1A1, DLL1, DTX1, IGF1, FGF3, FZD1, JAG1, KRT15, MSX1, NCAM1 *and *NOTCH1*. Of note, many of these genes were related to the stem cells self-renewal, regulation of symmetric/asymmetric division and pluripotency.

**Figure 6:**
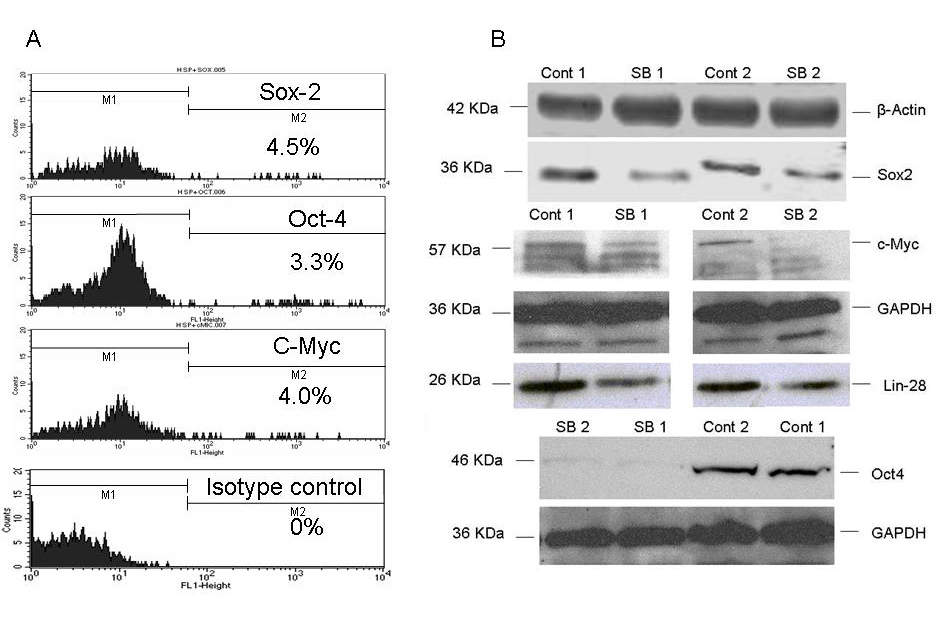
**Drug-induced alterations in the expression of the markers of pluripotency**. FACS analysis shows the presence of minor subpopulations of colon cancer cells within the 3D spheroids induced by CD133^high^/CD44^high ^cells (A), which express the three key pluripotency genes (Sox2, Oct4, and c-Myc). Western blot analysis shows the SB-T-1214-induced changes in the levels of protein expression of these pluripotency markers (B). Untreated spheroids (Con 1 and Cont 2); treated with drug (SB 1 and SB 2).

We have found that the colonospheres induced by HCT116 cells with CD133^high^/CD44^high ^phenotype contained minority cell populations with high levels of expression of several markers, which are essential for pluripotency and self-renewal of embryonic stem cells, including Oct4, Sox2, Nanog and c-Myc (Figure 6A). To analyze possible drug-induced alterations in the expression of these stem cell-specific transcription factors, which are low in abundance and present in a minority of colon cancer cell populations, we treated floating spheroids with 100 nM of SB-T-1214 for 24 hours to induce such alterations, but avoid profound cell death. Importantly, both FACS and western blot analyses have shown that the expression of these key pluripotency markers was suppressed even after single treatment with relatively low drug concentration (Figure 6B; western blot analysis is shown). Off note, we have determined that GAPDH expression was stable in all cell phenotypes, in contrast to other housekeeping genes, which is in line with recent report [53].

## Discussion

Increasing evidence indicates that effective anticancer drugs should target cancer-specific tumor-initiating cells, which are functionally and morphologically different from their bulk tumor counterparts. In our previous studies we found that in several analyzed clinically aggressive metastatic colon cancer specimens, as well as in the established colon cancer HCT116 cell line derived from a poorly-differentiated colonic adenocarcinoma, the majority of cells expressed low-to-moderated levels of CD133, CD44 and CD166 [20]. However, FACS analysis clearly showed the presence of the minority cell population with highest levels of combined expression of the CD133 and CD44. In contrast to the major bulk cell population, CD133^high^/CD44^high ^phenotype possessed high tumorigenic and clonogenic capacities after serial transplantations of the low cell numbers, and was able to produce cells of all the original phenotypes, showing characteristic stem cell plasticity [54]. Original cell phenotypes were retained in both mice tumor xenografts and floating colonospheres induced by CD133^high^/CD44^high ^cells, suggesting that they represent the tumor-initiating, or colon CSCs in a HCT116 cell line. Moreover, both mice tumors and spheroids induced by highly purified CSCs contain higher ratios of cells with original transplanted phenotypes compared to original sources, which is in line with other studies [55]. Genome-wide gene expression analysis with the Affymetrix DNA microarray assay revealed that in a CD133^high^/CD44^high ^cell population, many genes related to drug resistance and stemness, including *NOTCH, Shh, Wnt, Oct4*, were significantly upregulated compared to their bulk counterparts [20]. In the present study we further characterized this colon CSC phenotype using pathway-specific gene expression profiling with PCR Array assay (SABiosciences) in the three independent colon cancer cell lines, HCT116, HT29 and DLD-1. We have found that CD133^high^/CD44^high ^cell phenotype was present in all these cell lines, possessed relatively high sphere-forming capacity, and induced spheroids which expressed upregulated levels of the majority of stem cell-related genes compared to their differentiated counterparts. However, HCT116 cell populations revealed significantly higher expression of the stemness genes and higher sphere-forming potential compared to HT29 and DLD-1.

There is accumulating knowledge that tumor-initiating cells in metastatic colon cancer may not be demarcated solely by the expression of CD133 [18,56]. Although both CD44 and CD133 were reported as putative markers for many cancer-specific CSCs, including colorectal cancer (described in Introduction), it remains to be resolved whether they are of equal functional importance and what are their interrelationships. A recent study has demonstrated the unique role of CD133 in the normal and malignant colon, showing that CD133^+ ^normal stem cells at the base of crypts in the adult intestine (a stem cell niche) not only generate the entire intestinal epithelium, but give rise to all the neoplastic cells in mice colon tumors [57]. It was shown that the proportion of CD133^+ ^cells in colon cancer metastases is higher than in primary tumors [58], which reflects the well known fact that metastatic lesions are more resistant to treatment. However, another study has shown that only a knockdown of CD44, but not CD133, strongly prevented clonal formation and inhibited tumorigenicity in mice xenograft model [59]. Authors reported that CD44^+ ^did not colocalize with CD133^+ ^cells within colorectal cancer. Similar results reported by Horst and colleagues showed that the expression of CD133 correlates with that of CD166, while both do not correlate with CD44 [60]. However, this data contradicts multiple reports which not only show colocalization of the CD133 and CD44 in several types of human cancer [13,17-20,61], but also suggest their combined expression as the best CSC marker [18,61]. Such inconsistency may be due to the high heterogeneity of clinical specimens, diversity of the experimental approaches, and lack of the specific CSC markers.

Therefore, we have selected the CD133^high^/CD44^high ^cell phenotype to study CSC-targeted activity of a new-generation taxoid, SB-T-1214 in three independent colon cancer cell lines. Given that this compound led to complete remission in *in vivo *colon cancer xenograft model (Pgp+ DLD-1), we hypothesized that it could modulate some stemness genes/signaling pathways. The traditional *in vitro *model, the monolayer of the bulk tumor cells, poorly represents normal physiological conditions and has limited relevance to the hierarchically organized *in vivo *tumors. In particular, this model is not optimal for stem cell-based studies, because even highly purified CSCs can undergo relatively fast differentiation after being placed in standard adherent cultures with serum-containing medium, and therefore, observed drug responses will be related to the bulk tumor cells, not CSCs. However, in our previous studies we have found that short-term culturing of repeatedly sorted cells on type I collagen-coated surfaces in serum-free stem cell medium led not only to the retaining, but to significant increase of the ratios of the tumor-initiating cell phenotypes [19,20]. This data is in line with a recent study showing that human colorectal carcinoma cells grown on type I collagen in serum-free medium undergo an epithelial-mesenchymal-like transition and downregulation of E-cadherin and β-catenin at cell-cell junctions [52]. Authors have found that collagen type I inhibited cell differentiation, increased clonogenicity and promoted expression of CD133 and Bmi1, indicating that it promoted expression of a stem cell-like phenotype in colon cancer cells. In this context, culturing the adherent to a type I collagen CSC-enriched cell population in a serum-free stem cell medium can provide a useful tool for the preliminary evaluation of the CSC-focused drug responses. However, altered cell-to-cell and cell-to-matrix contacts in monolayer cell cultures can affect their signal transduction pathways and response to treatment [62-65]; therefore, we studied the SB-T-1214 cytotoxicity using an alternative model: free-floating colon cancer spheroids induced by the purified colon cancer tumorigenioc cell phenotypes. A three-dimensional model of cancer spheroids was established by Sutherland and colleagues [66,67] more than three decades before the discovery of CSCs in human cancers. It is now well documented that this model is more closely related to original tumors with respect to cell morphology, metabolic and proliferative gradients, oxygen and drug penetration and cell-cell junctions compared to the traditional cancer cell monolayers [66-70]. In addition, cancer spheroids are organized hierarchically, similarly to the *in vivo *tumors, containing a relatively small population of the tumorigenic cells and a large spectrum of their progenitors and differentiated cells, which comprise a bulk mass of the tumor or spheroid [70-72]. Spheroid cultures favor the proliferation of undifferentiated cells and can be passaged for many generations, reflecting the fact that they contain a population of cells with profound self-renewal capacity. We have shown that both mice tumor xenografts and 3D spheroids induced by more purified CD133^high^/CD44^high ^cells contained much higher ratios of cells with the original phenotype, and possessed higher tumorigenic and clonogenic potentials [19,20]. Similar findings were reported for metastatic breast cancer cells [73] and prostate cancer cells lines [16]. Many of the commonly used anti-cancer drugs induce about 20-fold lower cytotoxicity against 3D cancer spheroids compared to monolayer cultures [74], and exhibit chemoresistance which recapitulates this resistant phenotype *in vivo *[39,62,74-78]. In this context, SB-T-1214 revealed promising cytotoxicity against CSC-induced floating colon spheroids. In contrast to earlier studies on 3D cell cultures, which were usually limited to the relatively short-term gross evaluation of the inhibition of spheroid growth and apoptosis, we have analyzed specific stem cell-related responses. Thus, using a stem cell-focused PCR array assay we have found that single treatment of the 3D spheroids induced by CD133^high^/CD44^high ^cell populations with relatively low concentrations of the SB-T-1214 resulted not only in significant down-regulation of the majority of stem cell-related genes, but more importantly, led to a dramatic reduction of their sphere-forming capacity. In addition, the expression of several key regulators of pluripotency of the embryonic stem cells, Oct-4, Sox-2, Nanog, Lin-28 and c-Myc, was also inhibited after single treatment with 100 nM of SB-T-1214 for 48 hours. The synergistic action of these tissue-specific transcription factors is a pivotal mechanism for determining cellular phenotypes and self-renewal of embryonic stem cells. Thus, the introduction of four genes (Oct-3/4, SOX2, c-Myc, and Klf4) into adult fibroblasts can transform them into pluripotent stem cells [50,51]. It has been demonstrated later that Oct-3/4 and SOX2 are crucial transcriptional regulators whose absence makes induction of pluripotency impossible, and additional genes, including Klf4, c-Myc, Nanog and LIN-28 are important for the induction efficiency [79]. There is growing data that the pluripotency markers are also expressed by CSCs. Thus, the CD133-positive cells isolated from lung cancer tissues possessed the characteristics of stem-like cells and malignant tumors and expressed Oct4 [80], which was linked to chemo- and radioresistant properties in lung cancer-derived CD133^+ ^cells. Expression of Oct3/4 and Sox2 was also associated with an unfavorable clinical outcome [81]. Of interest, down-regulation of Lin-28 can lead to the rapid production of the mature Let-7 g miRNAs, which are required for cell differentiation [82]. Therefore, significantly decreased levels of expression of stem cell-related genes in general, and shut-down of several major players, including Oct-4, Sox-2, c-Myc and Lin-28 after treatment with SB-T-1214 is promising, because it most likely means that treated CSCs in colonospheres were promoted to a more differentiated state. This data is important in the light of growing evidence indicating that standard anti-cancer therapies often promote self-renewal of tumor-initiating cells and further resistance to treatment [28-30]. CSC resistance to treatment may be due to several mechanisms, including overactivated ABC transporters, high capacity for DNA repair and activated anti-apoptotic machinery [43-45]. It was recently demonstrated that treatment with 5-FU and oxaliplatin, a standard therapy for metastatic colon cancer, induced up to 30-fold enrichment of CD133+ and up to 2-fold enrichment of CD44+ cells in HT29 cell line [31]. This data is in line with our observation that after a single treatment with 100 μM Paclitaxel for 24 hr, the clonogenic potential of the dissociated HT29 and DLD-1 spheres cells was significantly increased, so we can assume that post-treatment spheroids contained a higher proportion of putative colon CSCs compared to untreated spheroids. Since we have studied the SB-T-1214 induced alterations in the stemness gene expression profiles using total cell lysates (equal amounts of the total RNA for PCR arrays and total protein for western blot analyses), the significant inhibition of the stem cell-related genes induced by SB-T-1214 is promising.

## Conclusions

In conclusion, we report here that a new-generation taxoid, SB-T-1214, possesses significant activity against 3D colon cancer spheroids induced by and enriched with drug resistant tumorigenic CD133^high^/CD44^high ^cell populations, and efficiently inhibits the expression of a majority of stem cell-related genes, including several key regulators of pluripotency and self-renewal of embryonic stem cells. Therefore, our data indicates that the long-term efficacy of SB-T-1214 against drug resistant colon tumors *in vivo *[49,83] may be explained by down-regulation of multiple stem cell-related genes in tumorigenic cell populations, in addition to known efficacy of taxoids as a mitotic poisons due to their binding to microtubules [84] in the proliferating pool of cancer cells. These findings should be further tested across a large series of clinical specimens of primary and metastatic lesions of colon cancer.

## Methods

### Cells and Culture systems

Standard HCT116 cells derived from a poorly-differentiated human colonic adenocarcinoma (ATCC) were maintained at SBU Cell Culture Facility. Before sorting, cells were grown in standard conditions (DMEM with 10% FCS, on uncoated flasks or dishes) at 50-75% confluency. Isolated candidate CSC phenotypic subpopulations of colon cancer cells were counted, resuspended in a serum-free Mesenchymal stem cell medium (MSCBM; Lonza, Walkersville, MD) and cultured in two different settings: either as adherent to a type I collagen (Biocoat; Becton Dickenson, Bedford, MA), or as free-floating spheroids.

### CSC Purification and Abs

Cells were labeled with one or several markers conjugated with different fluorescent dyes, including anti-human CD133/2-APC (clone 293C3; Miltenyi Biotec, CA, USA); CD166-PE (clone 105902; R&D Systems, MN, USA); CD44-FITC (clone F10-44-2), CD44-PE (clone F10-44-2; Invitrogen/Biosources, USA); CD44v6-FITC (clone 2F10; R&D Systems, USA), *h*EpCAM-FITC (Biosource, CA, USA). Antibodies were diluted in buffer containing 5% BSA, 1 mM EDTA and 15-20% blocking reagent (Miltenyi Biotec, CA, USA) to inhibit unspecific binding to non-target cells. After 15 min incubation at 4°C, stained cells were sorted with multiparametric flow cytometry with BD FACS *Aria *cell sorter (Becton Dickinson, CA) in sterile conditions.

For ***immunohistochemical analysis **h*EpCAM-FITC (Biosource, CA, USA), biotin-conjugated anti-human CD133 as primary Abs (Miltenyi Biotec, CA, USA) and streptavidin-FITC (BD Pharmingen, USA) as a secondary Abs were used.

#### Generation of floating multucellular spheroids

Four hunderd cells of particular phenotype were seeded on each well of the Ultra-Low Attachment (ULA) 6-well plate (Corning, Lowell, MA) in serum-free MSCB medium containing 10-25% Matrigel matrix (BD Biosciences) and examined after 1 week of culturing under standard conditions. Fresh medium was added after one week of culturing, every third day.

#### Sphere propagation

After initial culturing during 1-2 weeks on ULA plates, primary spheres were gently disaggregated by repeated pipetting and transferred into ULA flasks for further propagation and maintenance.

#### Sphere formation assay

Clonogenic potential of different phenotypic subpopulations was determined before and after treatment with SB-T-1214. Cells were counted, resuspended in MSCBM/Matrigel and plated on 48-well plates at a final count of 300 cells per well. One to two weeks after initiation, the plates were inspected for colony (sphere) growth, the number of colonies within each well was quantified by phase contrast microscopy and representative fields were photographed.

Isolation of the CSC populations from peritoneal wash of colon cancer patients and solid tumors, as well as induction of the mouse tumor xenografts, were carried out as we described elsewhere [20].

### Drug Treatment

#### In vivo cytotoxicity

All experiments involving the use of animals were performed in accordance with SBU institutional animal welfare guidelines. Initially, the SB-T-1214 was evaluated for its efficacy against a drug-resistant human colon tumor xenograft (Pgp+) DLD-1 in severe combined immune deficient mice (SCID). Taxoid was administered intravenously at three doses 3 times using 3 day regimens (q3d × 3, on day 5, 8, and 11), starting from day 5 after DLD-1 subcutaneous tumor implantation.

#### Cell death

was analyzed with flow cytometry using either Annexin V/PI staining (BD PharMingen) or Live/Dead assay (BioVision, CA) as recommended by the manufacturers.

#### In vitro cytotoxicity

was studied in two settings: a) using adherent type-I collagen monolayer cultures of freshly isolated CD133^high^/CD44^high ^cells; and b) three-dimensional cultures of floating multicellular spheroids induced by CD133^high^/CD44^high ^and CD133-negative cells.

#### Adherent to type I collagen cultures

The CD133^high^/CD44^high ^cells (4-5 × 10^3 ^cells/well were seeded onto the type I collagen-coated 6-well plates, and the experiments were initiated 24 h later, upon sub-confluency. The regular MSCB medium was replaced with treatment media containing SB-T-1214 at selected concentrations (0.001-0.1 μg/ml). Treatment media was removed 24 hr later, followed by washing with regular MSCBM and dissociation of cells with an Enzyme-free dissociating reagent (Chemicon International). Upon termination (48 hr from start), cell viability and cell death was analyzed through flow cytometry.

### 3D culture

Cytotoxicity studies were carried out when floating spheroids induced by different cell phenotypes reached about 400-500 μm in diameter. Spheres were resuspended in the drug-containing MSCBM (0.01-0.1 μg/ml) and either plated on ULA 48-well plates (5-10 spheres/well) for microscopy or remained in ULA flasks if a larger number of sphere cells was required for FACS, PCR array, western blot and other analyses. After treatment with the drug for 48 hours, spheroids/cells were rinsed twice in PBS, centrifuged for 5 min at 1,000 rpm, and incubated in regular MSCBM for 24 hr. Then, rinsed spheroids/cells were dissociated with an Enzyme-free dissociating reagent and single cell suspensions were further analyzed.

### PCR ArrayAssay

Stem cell-specific gene expression profiles were studied with the PCR Array assay (SABiosciences, CA) in accordance with the manufacturer's recommendations. Briefly, total RNA was isolated from different cell populations or whole floating spheroids using PARIS kit (Ambion). Up to 1 μg of total RNA was treated with DNase and cDNA was prepared using RT^2 ^First Strand kit. For each analysis, pairs of the test and control cDNA samples were mixed with RT^2 ^qPCR Master mix and distributed across the PCR array 96-well plates, each of which contained 84 stem cell-related probes and control housekeeping genes. After cycling with real-time PCR (Opticon MJ Research or ABI 7300, Applied Biosystems), obtained amplification data (fold-changes in C_t _values of all the genes) was analyzed with SABiosciences software.

### Western blotting

Cell pellets were suspended in Lysis Buffer (Active Motif, CA) and incubated for 10 min on ice on a rocking platform. After brief vortexing, cell lysates were centrifuged at 4,000 g for 30 min at 4°C. The protein content in the supernatant was determined by a Bradford method, and equivalent amounts of total proteins (10 μg) were resolved on 10% SDS-PAGE gel. After transferring to a polyvinylidene fluoride membrane, levels of various proteins were determined by Western blot analysis using antibodies specific for Oct4, Sox2, Nanog, Lin-28, c-Myc, GAPDH and β-actin, respectively (1:500 dilution, 4°C, overnight). Following incubation with peroxidase-conjugated secondary Abs (anti-rabbit IgG; ECL, UK) for 1 hr at 25°C, blots were developed using the enhanced chemiluminesens (ECL) reagents. Alternatively, the blots were washed three times with PBST and incubated with AlexaFluor 680-conjugated goat anti-rabbit secondary antibody (Invitrogen) for 1 hr. Blots were then washed three times with PBST, twice with water, and the image captured on an Odyssey Infrared Imaging System (Li-Cor Biosciences).

#### Statistical analysis

The statistical significance of differences was determined using Student's *t*-test. *P *< 0.05 was considered statistically significant.

## List of abbreviations

CSCs: cancer stem cells; MSCBM: mesenchymal stem cell basal medium; SB-T-1214: new-generation taxoid; ULA: ultra low attachment plates.

## Competing interests

The authors declare that they have no competing interests.

## Authors' contributions

GIB conducted and carried out this project, reviewed and evaluated all the experimental data. YW, MD, EZ and JJ participated in PCR array analysis. AS, HW and SZ carried out western blotting. YL participated in sphere formation assay and data analysis. RR was responsible for cell culture. KS provided a histopathological evaluation of the mice tumor xenografts. All authors red and approved the final version.
